# Transactions of the American Medical Association, Vol. XVIII

**Published:** 1867-11

**Authors:** 


Transactions of the American Medical Association, Vol XVIII. Philadelphia: Printed for the Association, by COLLINS, 705 Jayne Street. 1867. Price $5.00

This, like the preceding volumes, is published in excellent style, and contains 551 pages. We have not time or space, at present, to enumerate the reports and papers it contains. We assure our readers, however, that they are worth more than the cost of the volume.

